# A Natural Lignification Inspired Super‐Hard Wood‐Based Composites with Extreme Resilience

**DOI:** 10.1002/adma.202502266

**Published:** 2025-03-27

**Authors:** Yuxiang Huang, Kaixin Jiang, Yingqi He, Juan Hu, Kirsten Dyer, Sherry Chen, Esther Akinlabi, Daihui Zhang, Xuehua Zhang, Yanglun Yu, Wenji Yu, Ben Bin Xu

**Affiliations:** ^1^ Research Institute of Wood Industry Chinese Academy of Forestry Beijing 100091 China; ^2^ Mechanical and Construction Engineering Northumbria University Newcastle Upon Tyne NE1 8ST UK; ^3^ Offshore Renewable Energy Catapult Blyth NE24 1LZ UK; ^4^ Institute of Chemical Industry of Forest Products Chinese Academy of Forestry Nanjing Jiangsu 210042 China; ^5^ Department of Chemical and Materials Engineering University of Alberta Edmonton T6G 1H9 Canada

**Keywords:** antipiercing performance, exceptional resilience, lignification inspiration, structural stability, wood‐based composites

## Abstract

The growing demand for high‐strength, durable materials capable of enduring extreme environments presents a significant challenge, particularly in balancing performance with sustainability. Conventional materials such as alloys and ceramics are nonrenewable, expensive, and require energy‐intensive production processes. Here, super‐hard wood‐based composites (WBC) inspired by the meso‐scale homogeneous lignification process intrinsic to tree growth are designed and developed. This hybrid structure is achieved innovatively by leveraging the infusion of low‐molecular‐weight phenol formaldehyde resin into the cell walls of thin wood slices, followed by a unique multi‐layer construction and high‐temperature compression. The resulting composite exhibits remarkable properties, including a Janka hardness of 24 382 N and a Brinell hardness of 40.7 HB, along with exceptional antipiercing performance. The created super‐hard, sustainable materials address the limitations of nonrenewable resources while providing enhanced protection, structural stability, and exceptional resilience. The WBC approach aligns with UN Sustainable Development Goals (SDGs) by offering extra values for improving personal safety and building integrity across various engineering applications.

## Graphical abstract

Super‐hard wood‐based composites (WBC) are designed and developed inspired by the mesoscale homogeneous lignification process intrinsic to tree growth. This innovative hybrid structure is achieved by leveraging the infusion of low‐molecular‐weight phenol formaldehyde resin into the cell walls of thin wood slices, followed by a unique multi‐layer construction and high‐temperature compression.

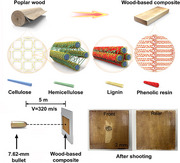

## 1 Introduction

The need for hard materials is increasing across various engineering fields because of their exceptional properties, including high hardness, strength, and durability. Materials like alloys, ceramics, and composites are essential in industries such as construction, automotive, and aerospace.^[^ ^1^ ^]^ However, the extensive utilization of traditional hard materials poses significant challenges, as they are often nonrenewable, costly to produce, and require energy‐intensive processes.^[^ ^2^ ^]^ The extraction and processing of raw materials, combined with the disposal of these products at the end of their life cycles, further contribute to their ecological footprint.^[^ ^3^ ^]^ For instance, the production of steel and concrete—two widely used hard materials—accounts for a considerable share of global CO_2_ emissions, underscoring the urgent need for more sustainable alternatives.^[^ ^4^ ^]^ This highlights the critical necessity of developing alternative materials that are not only environmentally friendly and cost‐efficient but also capable of meeting the stringent performance demands of modern engineering applications.^[^ ^5^ ^]^

Wood, a material with a long history of use in human civilization, presents a promising solution to address modern sustainability challenges.^[^ ^6^ ^]^ For thousands of years, it has been employed in construction,^[^ ^7^ ^]^ furniture,^[^ ^8^ ^]^ and tools, demonstrating its versatility and reliability as a structural material.^[^ ^9^ ^]^ With over three trillion mature trees on Earth, wood represents an abundant and renewable resource for a wide range of applications.^[^ ^10^ ^]^ Its natural abundance and renewability have positioned wood and its derivatives as attractive, low‐cost alternatives to petroleum‐based materials like plastics and nonsustainable materials such as concrete and steel.^[^ ^11^ ^]^ Compared to traditional materials, wood offers advantages including lower energy requirements for production and a reduced carbon footprint.^[^ ^12^ ^]^ The primary component of wood, cellulose, comprises 40–45% of its weight and is the most abundant biopolymer on Earth, offering exceptional intrinsic mechanical properties.^[^ ^13^ ^]^ For instance, cellulose has a stiffness of 150 GPa and a theoretical tensile strength ranging from 1.6 to 7.7 GPa.^[^ ^14^ ^]^ Its low density (1.5–1.6 g cm^−^^3^) further enhances its specific strength,^[^ ^15^ ^]^ which ranges from 1.0 to 5.1 GPa cm^3^ g^−1^, outperforming many engineering materials, including titanium alloys.^[^ ^16^ ^]^

With its highly desirable properties, wood shows immense potential to be a high‐performance structural materials. Traditional techniques to enhance wood's hardness typically involve delignification followed by compression densification, where Song et al. applied such an approach to obtain a material with a specific hardness up to 30 folds greater than natural wood.^[^ ^17^ ^]^ Similarly, Chen et al. showed that directly compressing natural wood could increase its hardness by 23 times, making it comparable to some metals.^[^ ^18^ ^]^ However, these approaches face challenges related to dimensional stability, as they depend on hydrogen bonding within the wood to maintain large deformations. This dependence can lead to swelling or warping, especially in humid or wet environments.^[^ ^19^ ^]^ Furthermore, the alkali treatment in delignification usually generates significant amounts of wastewater, raising environmental concerns.^[^ ^20^ ^]^

**Figure 1:**
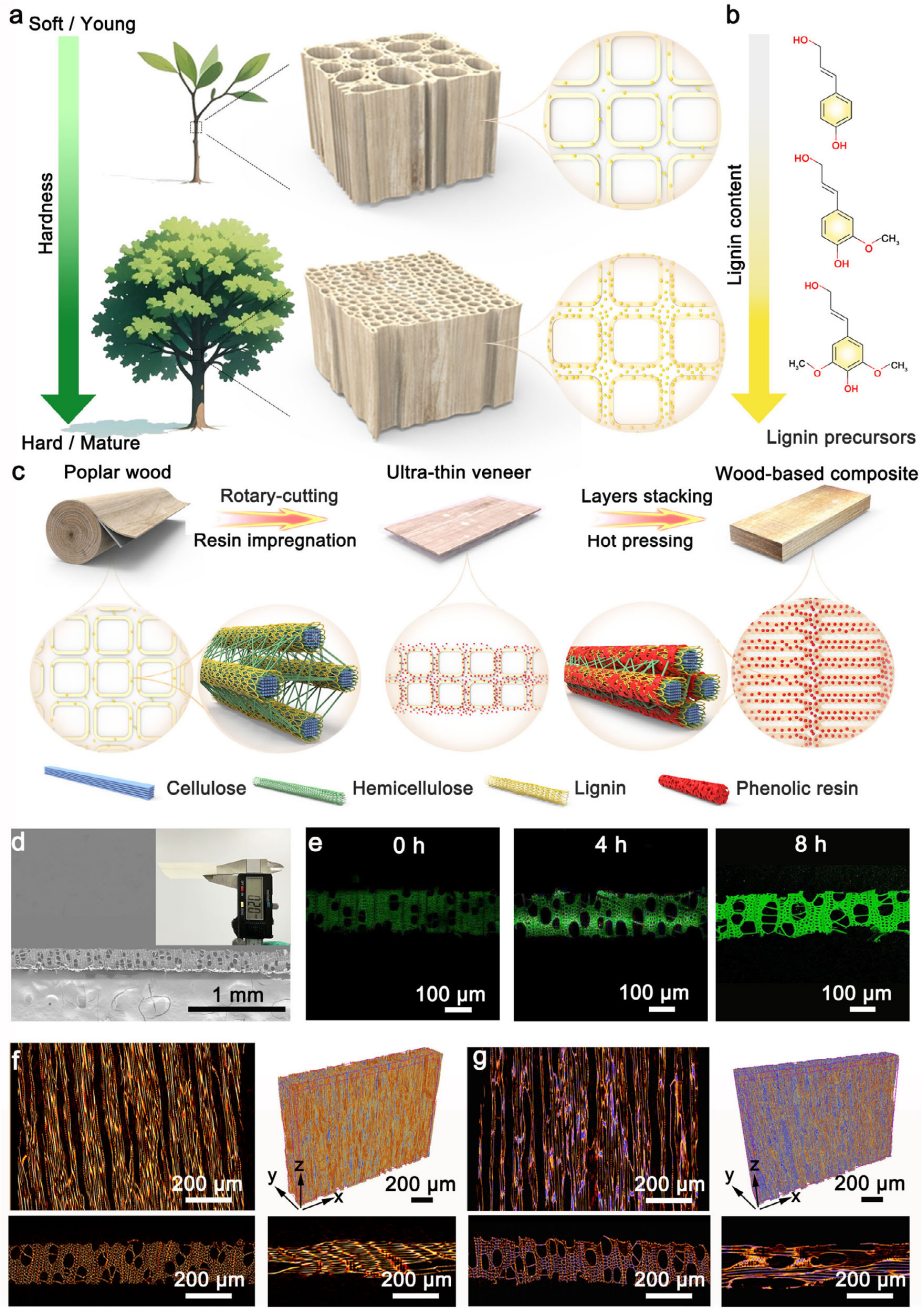
Schematic of the natural lignification and its inspired fabrication of WBC. a) The illustration of the lignification process during tree growth. b) Chemical structure of lignin: from top to bottom, the *p*‐coumaryl alcohol (H), coniferyl alcohol (G), and sinapyl alcohol (S) units. c) Schematic of the fabrication of WBC. d) Microscopic observation of poplar wood veneer (0.2 mm in thickness). e) LSCM images of poplar wood veneer after resin impregnation; Micro‐CT images of poplar wood veneer before f) and after resin impregnation g).

The natural growth of wood, from a small seedling into a strong, towering tree, involves a remarkable transition from a soft to a hard material (**Figure** **1**a). This transformation is largely driven by lignification, during which the lignin is deposited around fiber bundles in a crust‐like manner. This deposition enhances the hardness of cell walls, enabling them to afford the tree's weight.^[^ ^21^ ^]^ As the tree matures, lignin deposition intensifies, increasing the wood's mechanical strength and rigidity.^[^ ^22^ ^]^ Concurrently, the thickening of cell walls, a reduction in the wall‐to‐lumen ratio, and an increase in substantive density further enhance the wood's structures, allowing it to endure substantial mechanical stress.^[^ ^23^ ^]^ Understanding this natural evolution offers valuable insights for developing advanced wood‐based materials.^[^ ^24^ ^]^

Drawing inspiration from the natural growth processes of trees and plants, this study presents an alternative approach to enhancing the hardness and structural integrity of wood. Unlike previous methods that can compromise environmental resistance and contribute to pollution,^[^ ^25^ ^]^ we develop super‐hard wood‐based composites (WBC) through the controlled infusion of small molecular weight phenol formaldehyde (PF) resin into the wood, followed by solidification. While PFs are widely applied in wood lamination, they mainly achieve surface‐level adhesion rather than intrinsic cell‐wall reinforcement—a key limitation addressed in this work. Our approach not only improves the hardness of cell walls but also enhances the overall structural stability of the material. The resulting material offers a novel, sustainable solution for various engineering applications, combining the renewable and eco‐friendly attributes of wood with the mechanical properties needed for modern engineering challenges.

## 2 Results and Discussion

### 2.1 Design Principle and Development of WBC

A typical maturing process of the tree, as illustrated in Figure 1a, begins with lignification. The phenolic polymer lignin, known for its cross‐linking properties, is deposited as a crust‐like material that encases fiber bundles, fulfilling the cell wall with the necessary hardness to support the plant's structure. Additionally, the cell wall thickens, reducing the wall‐to‐lumen ratio and increasing parenchymal density. Inspired by this natural process, we infuse low molecular weight phenol formaldehyde (PF) resin into the cell walls to enhance the content and density of crust‐like structures. The resin infiltrates and solidifies, increasing the hardness of cell walls (Figure 1b; Figure S1, Supporting Information). During the thermal curing process, the PF resin also acts as a plasticizer, enabling further compression and compaction of the wood. This compaction increases the wall‐to‐lumen ratio, enhances solid density, and strengthens interactions within the cellulose skeleton, leading to super‐hard WBC (Figure 1c).

Ultra‐thin veneers offer significant advantages by enabling complete resin impregnation while preserving the integrity of cell walls during compression. The wood veneer used in this study has a thickness of 0.2 mm (Figure 1d), approaching the minimum achievable via rotary cutting. SEM images reveal the presence of 4–5 conduits or over ten wood fiber cells in the thickness direction. Owing to the capillary effect and osmotic pressure within the mesoporous structure, the resin exhibits excellent wetting properties on the veneer's surface and rapidly penetrates it (Figure S2, Supporting Information). The fluorescence of PF resin outlines its penetration depth under laser confocal scanning microscopy (LCSM). After 8 h, the entire veneer presents bright green (Figure 1e), contrasting with the dark green of the veneer derived from fluorescent lignin (Figure S3, Supporting Information), confirming the complete impregnation. This is further supported by micro‐CT images of poplar wood veneer (Figure 1f,g), which show a uniform distribution of resin throughout the veneer. However, the resin penetration achieves limited progress with a thicker veneer (Figure S4, Supporting Information, 6 mm in thickness), only the surface gets a bright green color (Figures S5 and S6, Supporting Information) after the same immersion period. This critical thickness threshold ensures resin penetration into cell walls rather than forming conventional adhesive layers.

### 2.2 The Homogenous Hybrid Structure and Mechanical Enhancement of WBC

**Figure 2:**
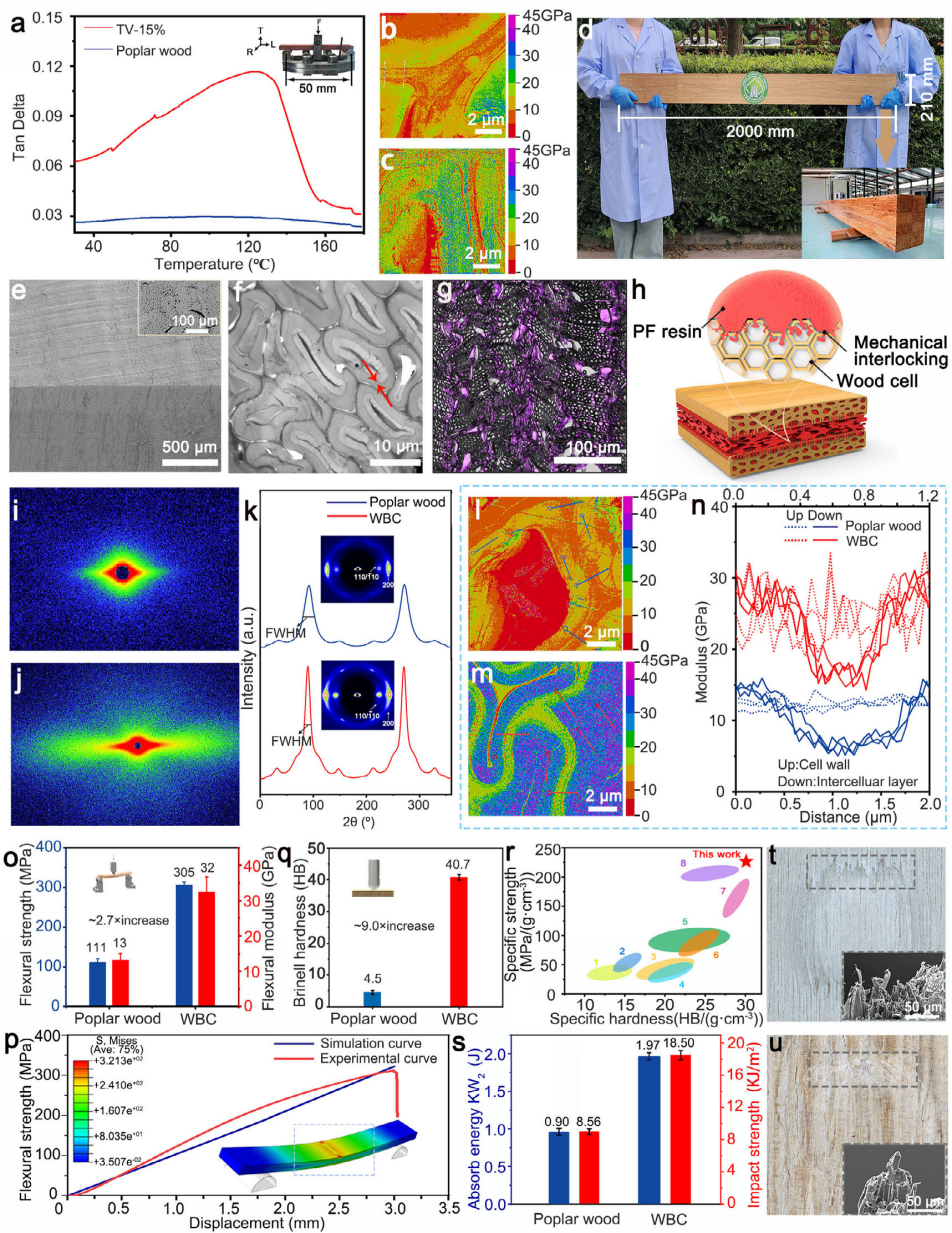
The multilength scale structural enhancement of WBC. a) Tan δ curves of the poplar wood and the TV‐15%. b,c) The modulus images of untreated poplar wood veneer b) and after impregnation and curing with PF resin c) measured by AFM (PF‐QNM). d) Presentation of super‐hard WBC with dimensions of 2000 mm × 210 mm × 20 mm. e) SEM images of the WBC. f) TEM images of WBC. g) LSCM images of WBC. h) Schematic of PF resin formed mechanical interlocks at different scales between veneer layers, inside cells, and inside cell walls. i,j) The SAXS pattern of poplar wood i) and WBC j). k) The 2D‐WAXD patterns of poplar wood and WBC. The azimuthal integration of the (200) scattering plane was employed to determine the microfibril orientation index (f_c_) of the samples. l,m) The modulus mapping images of poplar wood l) and WBC m) measured by AFM (PF‐QNM). n) Cell wall and intercellular layer modulus distribution curves along the dotted line in modulus mapping images of poplar wood and WBC. o) Flexural strength and modulus of the poplar wood and WBC. p) Comparison of experimental and simulated bending stress‐displacement curves for WBC. q) Brinell hardness of the poplar wood and WBC. r) Specific Brinell hardness versus specific strength Ashby plots for WBC, some alloys, and engineering materials. 1‐WPC, 2‐Q235 Steel, 3‐Stainless steel SUS316L, 4‐PMMA, 5‐GFRP, 6‐Al alloy A360, 7‐Mg alloy AZ61, 8‐CFRP s) Absorb energy and impact strength of poplar wood and WBC. t,u) UDTM images after hardness‐induced fracture morphology of poplar wood and WBC.

The use of low molecular weight PF resin with excellent fluidity at high temperatures and structural compatibility to wood is critical, allowing it to penetrate wood cell walls effectively and harden them upon curing. In contrast, melamine‐modified urea‐formaldehyde resin (MUF) was unable to fully impregnate the veneer, due to its higher viscosity and structural dissimilarity to wood (Figure S7, Supporting Information). Moreover, the MUF‐impregnated thick veneer show a highly inhomogeneity on resin distribution, which causes cell walls damaged during the hot pressing(Figure S8, Supporting Information). The Dynamic Mechanical Analysis (DMA) results show that thin veneers impregnated with 15 wt.% PF resin (TV‐15%) exhibit higher viscosity than untreated poplar wood at high temperatures, demonstrating improved plasticity due to PF resin (**Figure** **2**a). This indicates that phenolic resin not only enhances impregnation but also softens the cell walls during hot pressing. Further testing shows that the modulus of untreated poplar wood veneer cell wall is ≈14 GPa (Figure 2b; Figure S9, Supporting Information), while the modulus increased to over 20 GPa after impregnation and curing with PF resin (Figure 2c; Figure S10, Supporting Information).

It should be noted that the composite layer made by 0.2 mm veneers expands from 3.18 mm to only 3.48 mm, reaching a thickness expansion of 9% after 24 h of water immersion. In contrast, composites made from 6 mm veneers, expanded to 8.26 mm— a thickness expansion of 160% (Figure S11, Supporting Information). Under identical conditions, the composites with MUF exhibited a thickness expansion rate of 35%, while those without any adhesive expanded by 161%, leading to disintegration (Figure S12, Supporting Information). Based on the structural homogeneity and stability during the process, we apply this method to successfully manufacture WBC with dimensions of 2000 mm × 210 mm (Figure 2d) to demonstrate its potential for scaling up industrial applications.

Figure S13 (Supporting Information) presents a two‐section SEM image of block‐shaped poplar wood, highlighting its high porosity of 58%, attributed to numerous conduits and cell cavities within its wood fiber (Figure S14, Supporting Information). After establishing the homogeneous hybrid structure, the density increases significantly from 0.40 to 1.35 g cm^−^^3^, compacting most conduits and wood cells (Figure 2e). During compression, the intercellular layer width of wood cells decreases from 0.99 ± 0.25 µm to 0.56 ± 0.13 µm (Figure 2f; Figure S15, Supporting Information). Laser Scanning Confocal Microscopy (LSCM) images unveil a uniform distribution of PF resin throughout the composite (Figure 2g). The resin formed mechanical interlocks across different scales—between veneer layers, within cells, and inside cell walls (Figure 2h). The interfacial bonding mechanism was further elucidated by FTIR spectroscopy (Figure S16, Supporting Information). The absence of new covalent bonds between PF and wood components indirectly supports the dominance of mechanical interlocking. Cellulose microfibres at the wood cell walls usually act as load‐bearing units during cell compression.^[^ ^26^ ^]^ By comparing the small‐angle X‐ray scattering (SAXS) results between poplar wood (Figure 2i) and WBC (Figure 2j), the WBC exhibits stronger equatorial stripe scattering patterns, suggesting an oriented arrangement of cellulose fibers for effective load‐bearing.^[^ ^27^ ^]^

We next assess the orientation index (*f*c) and crystallinity index (*CI*) of samples using wide‐angle X‐ray diffraction (WAXD). Compared with the poplar wood (0.89, Figure 2k), the 2D‐WAXD pattern for WBC exhibits a higher microfibre orientation index of 0.91, suggesting more fibers align with enhanced interactions to increase the stiffness.^[^ ^28^ ^]^ The 2D‐WAXD pattern of poplar wood reveals the typical cellulose I β crystal structure. The structure of wood material remains unchanged after resin impregnation and hot pressing (Figures S17–S18, Supporting Information), and the crystallinity slightly increased from 51.74% to 55.94%. This increase stems from augmentation or rearrangement of crystalline regions during the high‐temperature and high‐pressure densification processes.^[^ ^29^ ^]^ This densely arranged, highly ordered, and highly crystalline cellulose structure at the micro‐level enhanced the mechanical properties of the material.^[^ ^30^ ^]^

The atomic force microscopy (AFM) was utilized to evaluate the interfacial modulus and visualize the mechanical properties. In the modulus graph, the red and orange areas indicate regions with low moduli, whereas the green and blue areas indicate high‐modulus regions (Figure 2l). The modulus graph reveals that both the cell wall (blue area) and intercellular layer (green area) of WBC experience enhanced modulus after compression and compaction (Figure 2m). Three modulus curves representing the cell wall and intercellular layer of both poplar wood and WBC were randomly selected from the modulus images (Figure 2n). In poplar wood, the cell wall modulus is ≈15 GPa, whereas the intercellular layer modulus is 5 GPa. Conversely, for WBC, the cell wall modulus ranges between 20 and 30 GPa, with the interlayer modulus over 15 GPa. The quasi‐static nanoindentation results demonstrate that the hardness and elastic modulus of WBC cell walls are 0.50 and 20.37 GPa, respectively, increases of 92.31% and 41.36% compared to poplar wood (Figure S19, Supporting Information).

The macroscopic mechanical properties of WBC, such as the static bending performance, tensile strength, and surface hardness, are characterized and analyzed. The static bending strength of poplar wood is 111 MPa, whereas WBC exhibit a strength of 305 MPa (Figure 2o) with an elastic modulus of 32 GPa. The commercial finite element software ABAQUS was employed to simulate the three‐point bending behavior (Figure 2p). The WBC board was modeled as linear elastic material with Young's modulus of 32 GPa, and the three pins were modeled as a discrete rigid body. The simulated flexural strength‐displacement curve suggests an agreement with the experimental data. In addition, the tensile strength increases from 48 to 243 MPa, a fourfold increase (Figure S20, Supporting Information), compared to the plain poplar wood.

In Figure 2q, poplar wood had a Brinell hardness value of 4.5 HB, while WBC demonstrated a significantly higher value of 40.7 HB, an eightfold increase. The Ashby diagram (Figure 2r) juxtaposes the specific hardness and strength of WBC with alloys and common engineering materials.^[^ ^31^ ^]^ This reveals that the specific Brinell hardness of WBC matches that of the magnesium alloy AZ61, surpassing those of metal materials such as Al alloy, stainless steel, and carbon steel, but also engineering materials like carbon fiber‐reinforced polymers (CFRP), glass fiber‐reinforced plastics (GFRC), polymethyl methacrylate (PMMA) and wood plastic composites (WPC). The Janka hardness of poplar wood and WBC are compared with other high‐quality hardwood materials (Figure S21, Supporting Information). WBC achieves a Janka hardness of 24382 N, marking a 19.5‐fold increase over poplar wood. Esteemed hardwoods, such as red sandalwood and snakewood, hold a Janka hardness of ≈16000 N, whereas WBC significantly surpass these hardwoods^[^ ^32^ ^]^ (Figure S22, Supporting Information). The metal pendulum impact test also shows that the impact toughness absorption energy and impact strength of WBC are 1.97 J and 18.50 KJ m^−2^, respectively (Figure 2s). The hardness of WBC can be tuned by changing material density and adhesive quantity (Figure S23, Supporting Information). This increase in hardness with higher density and adhesive quantity is attributed to enhanced structural compaction and interfacial bonding. As the density increases, the porosity within the material decreases, leading to a more compact and continuous structure that better resists deformation. Simultaneously, higher adhesive quantity ensures more complete infiltration and cross‐linking within the wood cell walls, forming a robust interlocking network that significantly improves load‐bearing capacity and hardness. In addition, unlike laminated composites where mechanical properties degrade with excessive layers, our cell‐wall‐level reinforcement eliminates interfacial dependency. The homogeneous hybrid structure (Figure 2h) ensures load transfer through resin‐cellulose interlocking rather than discrete adhesive zones, explaining its superior mechanical properties.

Fiber pullout, debonding, and fracture represent the typical failure modes in fiber‐reinforced composites.^[^ ^33^ ^]^ In this case, poplar wood displays dislocation fractures around the indentation (Figure 2t) and fiber pull‐out and interface debonding (Figure S24, Supporting Information) under an indentation load owing to the weak interfacial bonding. Conversely, around the WBC indentation, the fractured section appears to be neat and compact (Figure 2u), with an explicit fracture observed in the fiber (Figure S25, Supporting Information). Moreover, conduits and wood fiber cells undergo complete collapse and are intertwined tightly with the PF resin in WBC, uniformly saturating the wood cells to facilitate effective load transfer among fibers and prevent fiber pull‐out induced by stress concentrations.^[^ ^34^ ^]^

### 2.3 The Kinetic Anti‐Piercing Performance of Super‐Hard WBC

**Figure 3:**
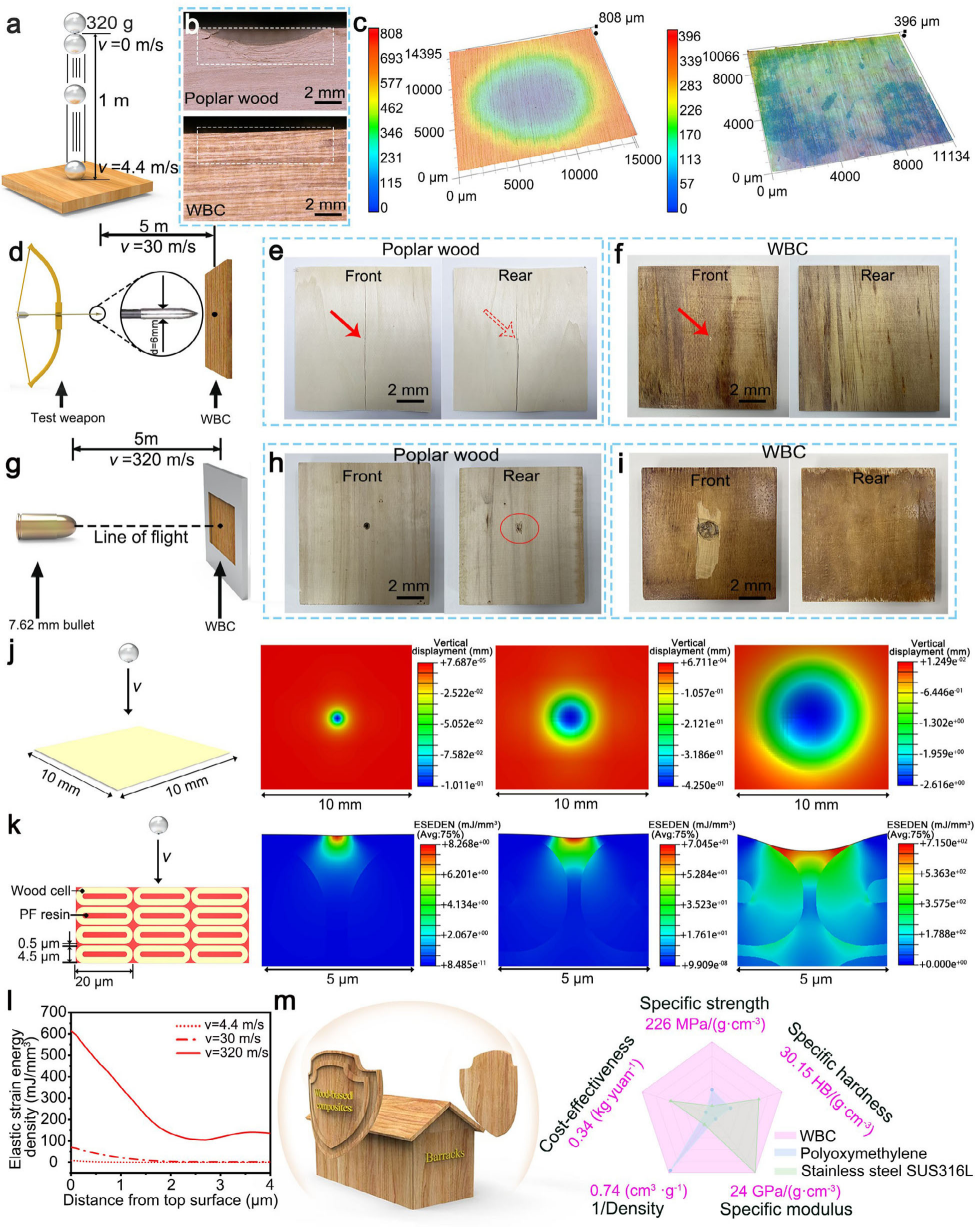
The kinetic anti‐piercing performance of WBC. a–c) The free‐falling ball experiment setup and results. d) The archery test with images on the front and rear of the target board made of e) poplar wood and f) WBC. g) The high‐speed bullet shooting test with the images on the front and rear of target board made of h) poplar wood and i) WBC. FEA simulation of impact with various incident KE on WBC with the j) top view, k) cross‐section view, and l) Deformation energy absorption versus distance from impact surface. m) The illustration of using WBC as shielding material, and a radar chart comparing with steel (stainless steel SUS3 16L) and engineering plastics (polyoxymethylene).

The kinetic anti‐piercing performance of WBC is further investigated by performing tests under various kinetic energy (KE) inputs, including free falling of a metal sphere at a relatively low speed (4.4 m s^−1^), archery test with an arrow speed of ≈30 m s^−1^, and gun shooting test with a bullet speed of ≈320 m s^−1^. In the free‐falling ball test, an aluminum sphere (mass ≈320 g) hit the sample surface (**Figure** **3**a) by free‐falling from a height of 1 meter (KE ≈3 J, impact radius ≈30 mm). The impact of the metal sphere creates an explicit dent on the surface of poplar wood, but not on the WBC surface (Figure 3b,c). At the archery test, the shooting distance was arranged as five meters with the terminal arrow speed measured at ≈30 m s^−1^ (KE ≈50 J, impact radius ≈0.5 mm) (Figure 3d). The poplar wood is completely pierced, with a clear crack to split the sample board (Figure 3e). In contrast, the WBC remained intact, showing only a minor impact dent by the steel arrowhead (Figure 3f). In the high‐speed bullet shooting test, a 7.62 mm model 1964 pistol was employed to fire from a distance of five meters to the sample board. With an incident velocity of ≈320 m s^−1^ (Figure 3g), the bullet easily hit through the 30 mm thick poplar wood (KE ≈510 J, impact radius ≈3.8 mm), leaving a distinct bullet hole on both the front and back (Figure 3h). When shooting at WBC, the bullet fails to penetrate the WBC board, a bullet hole is observed at the front of the board, while the back surface remains undamaged (Figure 3i).

To understand the impact mechanics, we simulate the impact process at the macroscale and microscale. Three impact velocities (4.4, 30, and 320 m s^−1^) were applied to the macroscale model, and the top‐view deformation profiles are shown in Figure 3j. The deformation profiles bring an explicit comparison on the surface hardness of the sample, which agrees well with the impact profile measured in Figure 3a. In the microscale model (Figure 3k), the average sizes of wood cells and the intercellular layer are created based on the data from Figure 2f. The side view plots reflect the distribution of impact energy under impact. The elastic strain energy density in the above numerical analysis is calculated in Figure 3l, which quantifies the capability of absorbing impact energy for the WBC sample board. For a sphere with an incoming speed of 320 m s^−1^, it can absorb 83.3% energy within the first 2 µm from the impact surface.

We also perform a puncture test by using a 6 mm needle to puncture sample at an approaching speed of 1 mm min^−1^. For poplar wood sample, the cracks appear at 30 s and penetrate the board through by 50 s (Figure S26 and Videos S1 and S2, Supporting Information). In contrast, no visible cracks are observed for WBC after 50 s. The maximum puncture force for poplar wood is recorded at 41.2 N, while a significantly higher resistance is obtained for WBC with a peak force of 180.1 N. Surface scratch resistance was assessed (Figure S27, Supporting Information) by employing a diamond needle to carve into the sample at a speed of 5 r min^−1^. Scratch marks are observed on the surface of poplar wood, while the WBC's surface presents no scratch. By comparing with other materials such as steel and engineering plastic (Figure 3m; Table S1, Supporting Information), WBC outperform them in a few properties, including specific hardness, specific strength, density, energy efficiency, and cost‐effectiveness.^[^ ^35^ ^]^ WBC achieves 8–12‐fold lower cost per m^3^ than metals and 3.6–14‐fold lower than engineered plastics, offering significant economic advantages for large‐scale construction.

### 2.4 The Environment Resilience of WBC

**Figure 4:**
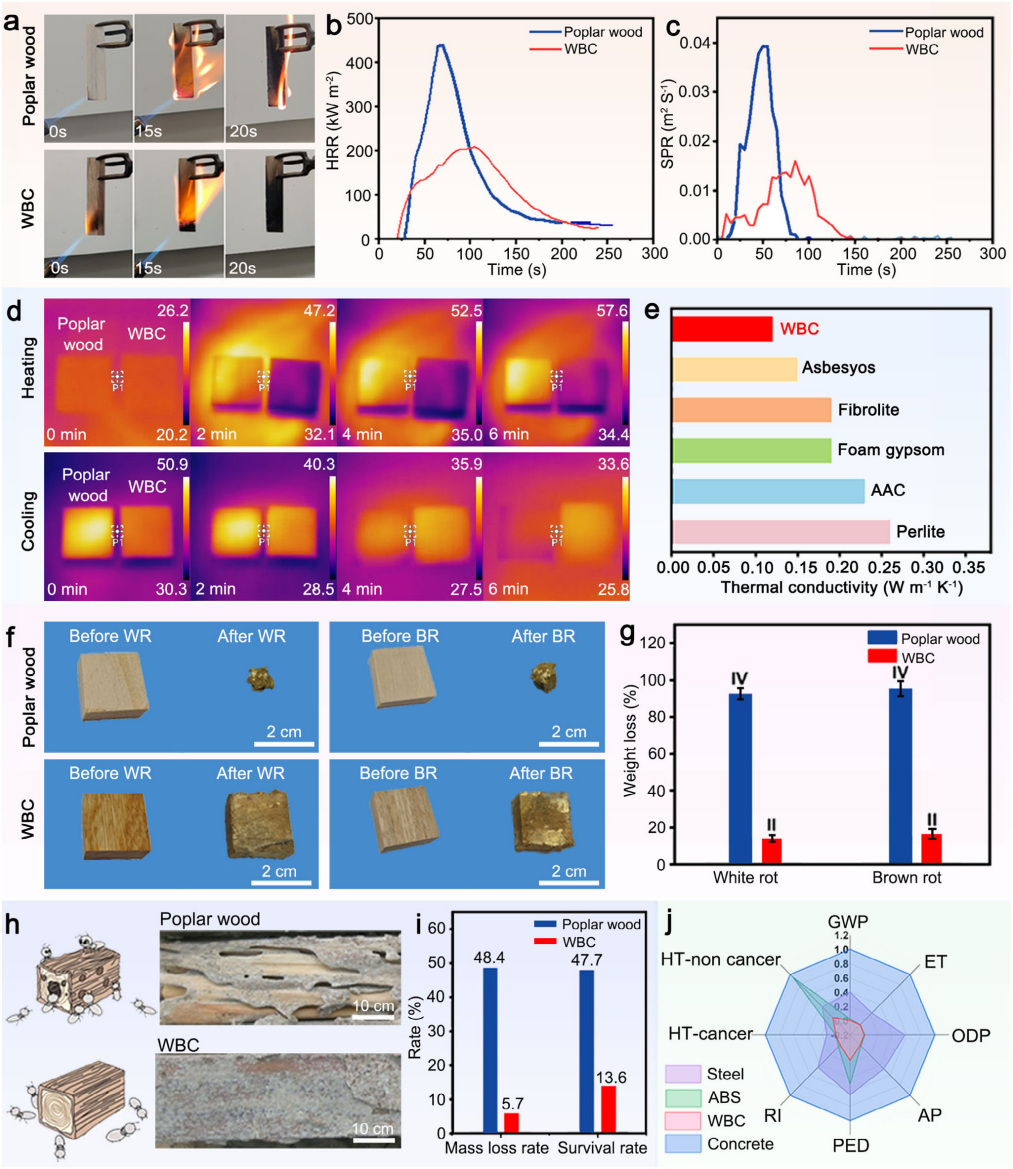
The environmental resilience of WBC. a) Comparison of flame‐retardant properties between poplar wood and WBC. b,c) HRR and SPR during combustion of the poplar wood and WBC. d) Infrared images of poplar wood and WBC. e) Thermal conductivity comparison of WBC and other materials. f) Comparison of corrosion resistance of poplar wood and WBC by WR (left) and BR fungi (right). g) Comparison of weight loss of poplar wood and WBC by white rot and brown rot fungi. h) Schematic diagram and physical images of termite resistance of poplar wood and WBC. i) Mass loss and survival rates of poplar wood and WBC. j) A comprehensive comparison between poplar wood and WBC in terms of human health carcinogenicity (HT‐cancer), noncarcinogenic toxicity (HT‐non cancer), respirable inorganic (RI), ecotoxicity (ET), global warming potential (GWP), acidification potential (AP), primary energy demand (PED), and ozone depletion potential (ODP).

We then assess the sample's features relating to its resilience such as flame retardancy, thermal conductivity, weather resistance and life cycle analysis. Compared to poplar wood, the WBC presents excellent flame retardancy after igniting for 15 s (**Figure** **4**a; Videos S3 and S4, Supporting Information). Cone calorimeter tests were conducted to simulate real fire scenarios and analyze the combustion characteristics for samples. The heat release rate (HRR), total heat release (THR), smoke production rate (SPR), and total smoke release (TSR) curves are presented in Figure 4b,c and Figures S28 and S29 (Supporting Information). The HRR of poplar wood is 439.4 kW m^−^^2^, while WBC exhibits a significantly reduced peak HRR of 208.9 kW m^−^^2^, a 52.5% decrease. The THR also decreases from 30.3 MJ m^−^^2^ for poplar wood to 24.6 MJ m^−^^2^ for WBC, representing a drop of 18.8%. Similarly, the peak SPR for poplar wood is 0.039 m^2^ s^−1^, whereas a maximum SPR of 0.016 m^2^ s^−^ is recorded for WBC, marking a significant reduction of 59%. While poplar wood reaches a total smoke production of 163.9 m^2^, WBC exhibits a value of 112.1 m^2^, which is 31.6% lower. The superior flame‐retardant performance of WBC stems from two synergistic mechanisms: char‐forming capability of PF resin and structural densification. First, PF resin inherently exhibits exceptional flame resistance due to its thermal decomposition behavior. During pyrolysis, PF generates a continuous, dense carbonaceous char layer that acts as both a thermal barrier and oxygen diffusion inhibitor, effectively suppressing flame propagation.^[^ ^36^ ^]^ Second, the high density (1.35 g cm^−^^3^) and resin‐cellulose interlocking network (Figure 2e) minimize internal porosity, reducing heat transfer pathways.

The thermal properties of samples were analyzed using an infrared imager to record thermal imaging under infrared light irradiation. Upon exposure to infrared light, poplar wood initially absorbs heat, and as the temperature continues to rise, the color transits from red to yellow and eventually to white (Figure 4d). The overall surface temperature of poplar wood exceeds 50 °C after 4 min. In contrast, WBC consistently displayed a purple hue, indicating its low apparent thermal conductivity. This is because the surface of poplar wood illuminated by the light source consists of tightly arranged conduits and fibers (continuous phase), with a thermal conductivity of ≈0.6 W m^−1^ K^−1^.^[^ ^37^ ^]^ In contrast, the WBC surface is a cross‐linked network (discontinuous phase) formed by wood cells and PF resin, which has a lower thermal conductivity of ≈0.15 W m^−1^ K^−1^. Additionally, WBC's low moisture content (5–6% vs poplar's 12%) minimizes water‐mediated heat transfer, further enhancing thermal insulation. During the cooling process, poplar wood dissipates heat quickly, completing this process in 6 min. Meanwhile, minimal heat diffusion is observed for WBC due to its low apparent thermal conductivity. The measured thermal conductivity of WBC is 0.12 W m^−1^ K^−1^, which is lower than that of several commercial insulation materials, including Asbesoys, Fibrolite, Foam Gypsum, autoclaved aerated concrete (AAC), and Perlite^[^ ^38^ ^]^ (Figure 4e).

The bio‐durability and lifespan of wood materials are important for sustainable development. After exposure to *Coniophora puteana* (white rot, WR) or *Gloeophyllum trabeum* (brown rot, BR) fungi for 12 weeks, only a small portion of poplar wood remains with 90% mass loss (Figure 4f). In contrast, WBC exhibits a quality loss of less than 25%, and decay resistance grade is elevated from Class IV to Class II (Figure 4g), demonstrating an enhanced resistance to fungal decay. This can be attributed to the low porosity (Figure S14, Supporting Information) limits water uptake, creating an unfavorable environment for fungal metabolism. The termite resistance was evaluated, where WBC demonstrates impressive performance. Different from the quality loss rate of 48.4% and survival rate of 47.7% in the poplar wood, WBC shows a significant reduction of 88.2% in average quality loss rate and 71.5% in termite survival rate (Figure 4h,i). Furthermore, the integrity level of these composites after termite exposure reached 9.0, indicating a significant advantage over poplar wood (Figure S30, Supporting Information). The exceptional termite resistance of WBC stems from its multi‐scale resin‐wood hybrid dense structure. The low porosity of WBC not only restricts water absorption, thereby inhibiting termite infestation, but its dense resin‐saturated matrix also effectively blocks termites' access to cellulose, their primary nutritional source.

We also carried out a life cycle assessment (LCA) to quantify its environmental impact compared to traditional materials such as engineering plastic (ABS), steel, and concrete. Our evaluation focused on eight environmental impact categories: HT‐cancer, HT‐non cancer, RI, ET, GWP, AP, PED, and ODP (Figure S31, Supporting Information). The results indicate that WBC have a lower environmental impact across all categories when compared to other structural materials^[^ ^39^ ^]^ (Figure 4j; Table S2, Supporting Information). To address formaldehyde emission concerns, the PF resin used in this study features a proprietary synthesis process designed to minimize formaldehyde release. Using this PF resin, the WBC achieved 0.007 mg m^−3^ formaldehyde release, below China's stringent Class ENF standard (0.025 mg m^−3^, GB/T 39600‐2021, Table S3, Supporting Information) and even lower than the formaldehyde released by poplar wood itself. Additionally, WBC demonstrates 0.01 mg m^−2^ h total volatile organic compound (T_VOC_), below the standard value of 0.05 mg m^−2^ h (T_VOC_, GB/T 39600‐2021, Table S4, Supporting Information). These findings underscore the significant role that WBC can play in mitigating global climate change, highlighting its potential as a sustainable and environmentally friendly material.

## 3 Conclusion

In summary, we design and develop novel super‐hard wood‐based composites (WBC), inspired by the natural lignification process. By infusing low molecular weight PF resin into wood cell walls, we realize a unique hardening mechanism from a meso‐structure level. The resulting WBC exhibits significant mechanical enhancement, including exceptional hardness and puncture resistance, outperforming untreated wood and even rivaling certain metals. Beyond mechanical superiority, these composites showcase exceptional resistance to surface scratches, fire, heat, decay, and termites, underscoring their robustness and suitability for demanding protective applications such as impact‐resistant panels and advanced structural materials. Importantly, the life cycle assessment (LCA) reveals that WBC provides significant environmental advantages, including substantial biomass carbon storage and a notably reduced global warming potential. By combining superior performance with environmental resilience, WBC represents a transformative solution that addresses critical challenges in modern engineering and materials science. This work demonstrates the potential for bio‐inspired, sustainable materials to redefine the landscape of high‐performance composites, paving the way for greener, more sustainable future applications.

## 4 Experimental Section

### Materials

Poplar wood (*Populus ussuriensis*), 5–6 years old, was acquired from the Heibei Province, China. The diameter of the trees at breast height was 20 cm, and the air‐dried density of the poplar wood was 0.40 g cm^−3^. The PF resin, developed by Taier Adhesive (Guangdong) Co., Ltd., possessed the following basic parameters: a solid content of 52.29%, molecular weights of 300 and 1500, and pH of 9.71.

### Preparation of Poplar Wood Veneer Prepregs

Poplar wood was truncated transversely to a length of 450 mm and then rotary cut parallel to the growth rings to obtain a thin veneer with a thickness of 0.5 mm. In a typical synthesis, the PF resin with a molecular weight of 300 was diluted to 13%, 18%, and 23% with water. Subsequently, the poplar wood veneers were soaked in a continuous impregnation device to incorporate the PF resin directly into the vessels, fiber lumen, and cell wall. The PF resin impregnation was conducted at 25 °C under ambient pressure for 8 h. To achieve the desired PF resin content (10%, 15%, and 20%), the poplar wood veneers were removed and leaked for 4 min to remove excess PF resin, and the resin content of the poplar wood veneer prepregs was calculated according to Equation (1).

(1) $$G_{2}=G_{1}+\frac{G_{1}\left(1-w\right)\times m}{P\times 100\%}$$

where *G*_1_ and *G*_2_ are the weights of the poplar wood veneer before and after immersion in the resin, respectively; *C* is the resin content; *P* is the solid content of the resin; and *w* is the moisture content of the poplar wood veneer before immersion.

### Fabrication of WBC

Poplar wood veneer prepregs were weighed based on final WBC densities (0.90, 1.00, 1.10, and 1.35 g cm^−3^). Subsequently, the weighed poplar wood veneer prepregs were symmetrically placed along the grain such that they were evenly and uniformly placed layer‐by‐layer in a 400 × 160 × 20 mm (L × W × H) mold to form a slab. In the pre‐compression stage, the slab was hot‐pressed by a hot press machine (CAR‐VER 3353, USA) at a closing speed of 1 mm min^−1^ at 150 °C, with the press closed for 6 min. Subsequently, the slab was compressed at 150 °C at 80 MPa for ≈20 min to obtain WBC.

### Characterizations

Ultradepth 3D microscopy (UDTM, VHX‐7000), scanning electron microscopy (SEM, Hitachi Regulus 8230), and transmission electron microscopy (TEM, Hitachi H‐750) were used to examine the surface morphology. The macroscopic distribution of the PF resin was observed using a laser scanning confocal microscope (LSCM Leica Microsystems, Bannockburn, IL, USA). Bruker Skyscan 1272 X‐ray micro‐computed tomography (µ‐CT) was applied to scan the fracture structure of samples. Mercury intrusion porosimetry (MIP, Micromeritics Instrument Corporation) was used to measure the porosity. 2D wide‐angle X‐ray diffraction (2D‐WAXD) was used to characterize the crystallinity (CI) and orientation index (fc) of the samples. Small‐angle X‐ray scattering (SAXS) was used to characterize the molecular arrangement of cellulose microfibrils. Fourier transform infrared (FTIR) spectra were obtained using an infrared spectrometer (VERTEX 80 V, Bruker, USA). A dynamic mechanical analysis (DMA, TA‐Q2980) was used to determine the viscoelasticity of the samples. Nano‐indentation (NI) test measurements were performed to determine the hardness and elastic modulus of the cell wall. Atomic force microscopy (AFM) measurements were performed in the PeakForce quantitative nanomechanical mapping (PF‐QNM) mode on a Bruker MultiMode AFM instrument (Bruker Dimension ICON). The middle lamella widths and fiber cell pore diameters were measured using the ImageJ software. Procedures are detailed in the Supplementary Information. The combustion properties of the samples were assessed using the vertical combustion method after 15 s of ignition. To simulate real fire conditions, a cone calorimeter (VOUCH, China) was employed to evaluate the combustion performance of the samples. Thermal conductivity (κ) was measured using a thermal conductivity analyzer (NETZSCH, Germany). Surface temperature changes were monitored by irradiating the samples with an infrared lamp and capturing the results with an infrared thermal imaging camera (IRay Tech, China). For termite resistance testing, samples measuring 25 × 25 × 6 mm were evaluated in accordance with GB/T18260‐2015 standards. Corrosion resistance was tested following the guidelines of GB/T 13 942.1‐2009. Formaldehyde and TVOC emissions were measured following GB/T 39600‐2021 using 1 m^3^ climate chambers. Untreated poplar controls were tested under identical conditions. Additionally, an LCA was conducted using Simapro software, focusing on categories such as global warming potential, acidification, ozone depletion, respiratory effects, and human health (including both carcinogenic and non‐carcinogenic toxicity), as well as ecotoxicity and primary energy demand. The functional unit for this assessment was defined as one kilogram of the composite material.

### Mechanical Tests

Janka hardness tests were conducted according to GB/T 1941–2021, using a hemispherical steel indenter with a diameter of (5.64 ± 0.01) mm, to push the indenter into the surface of the samples (50 mm × 50 mm × 15 mm) at a speed of 3 mm min^−1^ and a depth of 2.82 mm. The Brinell hardness was measured using a Brinell hardness tester (Shanghai Shangcai, XHB‐3000Z/CCD), and the test conditions for WBC (50 mm × 50 mm × 15 mm) included a 2.5 mm carbide indenter, 62.5 kg load, and 25 s holding time. Tensile tests were performed on the samples carefully cut into dumbbell shapes with a neck contraction area measuring 0.8 × 25 mm (T × L) using a universal mechanical testing machine (ETM 105D, Shenzhen Wance) at a rate of 1 mm min^−1^. Three‐point bending tests were conducted on the bulk samples with dimensions of 100 mm × 15 mm × 3 mm using the same universal mechanical testing machine (ETM 105D, Shenzhen Wance) at a rate of 2 mm min^−1^ with a support span of 60 mm. Width swelling rate (WSR), thickness swelling rate (TSR), and water absorption rate (WAR) of the specimens were measured according to the GB/T 30364–2013. Specimens (50 × 50 × 13 mm) were boiled at 63 °C for 24 h.

### Numerical Simulation

The commercial simulation package was used—ABAQUS, to simulate impact test and deformation on the surface of sample board subject to the hit of a sphere with different speeds. For both models, the WBC board was treated as a linear elastic material with Young's modulus of 32 GPa and a density of 1.35 g cm^−^^3^. Three impact velocities (4.4, 30, and 320 m s^−1^) were set and the distribution of maximum displacement was plotted. In the microscale model, PF and wood cells were modeled as linear elastic materials with elastic moduli of 15 and 30 GPa, respectively, and a density of 1.35 g cm^−^^3^. The geometrical inputs have been magnified due to the limitation of mesh size in ABAQUS. An element type CAX8H was used for mesh.

## Conflict of Interest

The authors declare no conflict of interest.

## Author Contributions

Y.X.H. and K.J. contributed equally to this work. Y.X.H., D.Z., Y.Y., and B.B.X. developed the concept. Y.X.H., Y.Q.H., J.H., and Y.Y. performed experiments and data acquisition. K.J., S.C., E.A., and B.B.X. performed the numerical analysis and FE simulation. All authors participated in the data analysis and the processes of figures and visualizations. The work was supervised by Y.Y. and B.B.X. The manuscript was written by Y.X.H., D.Z., S.C. X.Z., Y.Y., and B.B.X., with contributions from all authors.

## Supporting information



Supporting Information

Supplemental Video 1

Supplemental Video 2

Supplemental Video 3

Supplemental Video 4

## Data Availability

The data that support the findings of this study are available in the supplementary material of this article.
