# Supplementary material for: A Natural Lignification Inspired Super‐Hard Wood‐Based Composites with Extreme Resilience
Source: Adv Mater. 2025 Mar 27;37(19):2502266. doi: 10.1002/adma.202502266 (PMC12075913; doi:10.1002/adma.202502266)
Supplement: Supplementary file 1 — Supporting Information [file ADMA-37-2502266-s004.docx]

**A Natural Lignification Inspired Super-Hard Wood-based Composites with Extreme Resilience**

*Yuxiang Huang ^1,⊥^, Kaixin Jiang ^2,⊥^, Yingqi He ^1^, Juan Hu^1^, Kirsten Dyer ^3^,*

*Sherry Chen ^2^, Esther Akinlabi ^2^, Daihui Zhang ^4^*, Xuehua Zhang ^5^*,
Yanglun Yu ^1^*, Wenji Yu^1^, Ben Bin Xu ^2^**

*^1^ Research Institute of Wood Industry, Chinese Academy of Forestry, Beijing 100091, China*

*^2^ Mechanical and Construction Engineering, Northumbria University, Newcastle Upon Tyne, NE1 8ST, UK*

*^3^ Offshore Renewable Energy Catapult, Blyth, NE24 1LZ, UK*

*^4^ Institute of Chemical Industry of Forest Products, Chinese Academy of Forestry, Nanjing, Jiangsu 210042, China*

*^5^ Department of Chemical and Materials Engineering, University of Alberta, T6G 1H9, Canada*

**^⊥^ Author has equal contribution**

*E-mail: [dhzhang@icifp.cn](mailto:dhzhang@icifp.cn) (D. Zhang); [xuehua.zhang@ualberta.ca](mailto:xuehua.zhang@ualberta.ca) (X.H. Zhang); [yuyanglun@caf.ac.cn](mailto:yuyanglun@caf.ac.cn) (Y. Yu); [Ben.xu@northumbria.ac.uk](mailto:Ben.xu@northumbria.ac.uk) (B.B. Xu);

**Video S1: Static puncture video of poplar wood**

**Video S2: Static puncture video of WBC**

**Video S3: Burning video of poplar wood**

**Video S4: Burning video of WBC**

**1. Experimental**

Ultra-Depth Three-Dimensional Microscope (UDTM, VHX-7000), Scanning electron microscopy (SEM, Hitachi Regulus 8230, the voltage of 5 kV) and transmission electron microscopy (TEM, Hitachi H-750, the acceleration voltage of 120 kV) measurements were employed to examine the surface morphology.

A slicer (RM2245, LEICA, Germany) was used to cut cross-sectional slices of 20 μm in thickness from poplar veneers impregnated with PF and MUF resin. After coloration for 5min in 0.5% toluidine blue solution, the sections were gradient dehydrated for 5min with 20%, 40%, 60%, 75%, 85%, 95%, 100% ethanol solution and then the film was sealed with glycerine. The macroscopic distribution of PF and MUF resin in poplar veneer was observed by laser scanning confocal microscopy (LSCM, Leica Microsystems Bannockburn IL). Images were reconstructed by means of the Leica confocal software. (Coherent, Santa Clara, CA, Germany).

Small samples measuring 3 mm × 3 mm × 0.2 mm were taken for testing. Micro-computed tomography (micro-CT, Skyscan1172, Bruker, Germany) was employed to observe the penetration and distribution of PF resin in poplar veneer by image analysis using Nrecon, Data Viewer, CTan, CTvol and CTvox software. A scaled image pixel size of 500 nm was adopted. Each sample was scanned with arotation step of 0.14°, taking 360° for a complete scan.

Mercury intrusion porosimetry (MIP, Micromeritics instrument Corporation, the maximum pressure of 413.76 MPa) measurement was applied to measure porosity. 2D-WAXD experiment was performed using a Rigaku Rapid II X ray with a voltage of 45 kV and a current of 66 mA. X-ray wavelength was 1.5409 Å. The sample was then transported to the detector at a distance of 69.16 mm. The orientation index (fc) could be calculated by Eq. (1) on the azimuth integral curves on 200 crystal planes, where FWHM denoted the azimuthal profiles' full width at half-maximum from the chosen equatorial reflection. For the crystallinity (*CI*), it was calculated as the ratio of the integrated intensity of the crystalline peaks to the sum of the crystalline and amorphous intensities following Eq. (2). The SAXS measurement was performed on a Xenocs (Xeuss 2.0) system equipped with a Cu Kα (λ = 1.54189 Å) microfocus source and a Detris Pilatus 300k detector. The sample was then transported to the detector at a distance of 1185 mm. Fourier transform infrared spectroscopy (FTIR) spectra was adopted for the samples utilizing an infrared spectrometric analyzer (VERTEX 80V, Bruker, USA). Dynamic mechanical analysis (DMA, TA-Q2980) measurement was performed on a specimen of about 55 × 10 × 4 mm^3^ (L × W × D) in three-point bending mode with a span of 50 mm at a frequency of 1HZ and a constant amplitude of 0.03 mm. The temperature ramp experiment was conducted by heating from 30°C to 180°C at a heating rate of 3°C/min. Nano-indentation (NI) test measurement was measured to measure hardness and elasticity modulus of cell wall. The target peak load was 200 μN, the loading−unloading rate was 50 μN·s^−1^ and the hold time of the peak load was 6 s. Atomic force microscope (AFM) measurement was utilized in the PeakForce quantitative nanomechanical mapping (PF-QNM) mode on a Bruker MultiMode AFM (Bruker Dimension ICON) under ambient conditions. Peak force was 270 nN. Modulus variations were collected over a randomly selected 10 μm × 10 μm surface area with a resolution of 256 × 256 pixels. The samples were not subjected to any embedding procedure to prevent unnecessary interference by the embedding medium. The middle lamella widths and fiber cell pore diameter were measured by Image J.

$fc=\frac{(180^{\circ}-FWHM)}{180^{\circ}}$ (1)

$CI=\frac{I_{crystalline}}{I_{crystalline}+I_{amorphous}}$ (2)

The drop-ball impact testing evaluated the impact interface of the specimen surface. A 320 g aluminum ball was dropped from a height of 1 meter onto the surface of both poplar wood and WBC. An Ultra-Depth Three-Dimensional Microscope was used to perform the 3D imaging of the pits.

A scratch tester (TQC Sheen SH0530, USA) was applied to evaluate the scratch resistance of the specimen surface. The dimensions of the specimen were 100 mm × 100 mm × 5 mm, and a load of 4 N was applied using weights, and the turntable was rotated at a speed of 5 r/min for 500 revolutions. An Ultra-Depth Three-Dimensional Microscope was adopted to image the scratches in three dimensions.


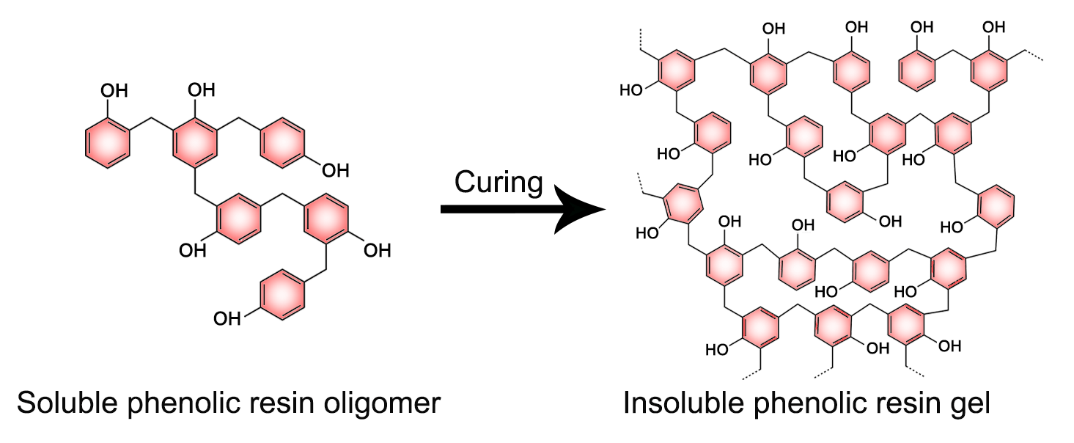


**Figure S1.** Schematic of phenolic resin preparation process.


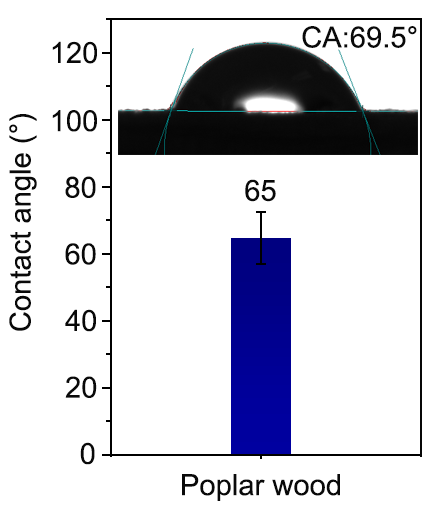


**Figure S2.** Surface contact angle test of phenolic resin on poplar wood surface.


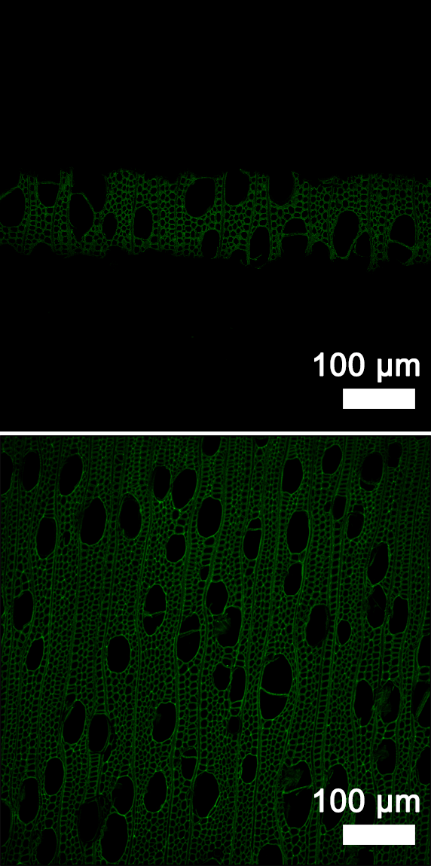


**Figure S3.** The LSCM images of the 0.2 mm thick poplar wood veneer.


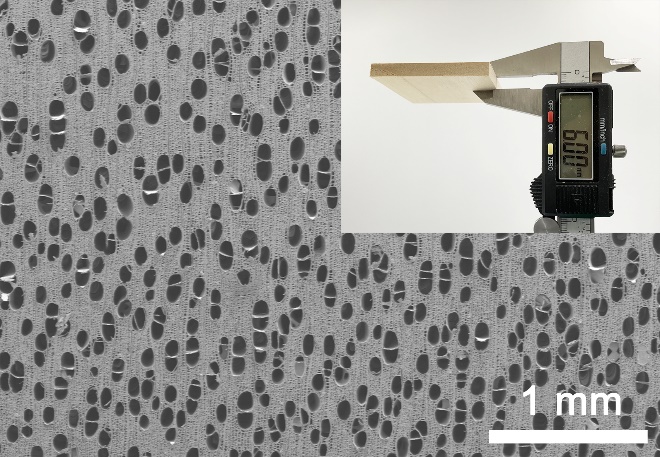


**Figure S4.** SEM image and corresponding physical image of poplar veneer with thickness of 6 mm.


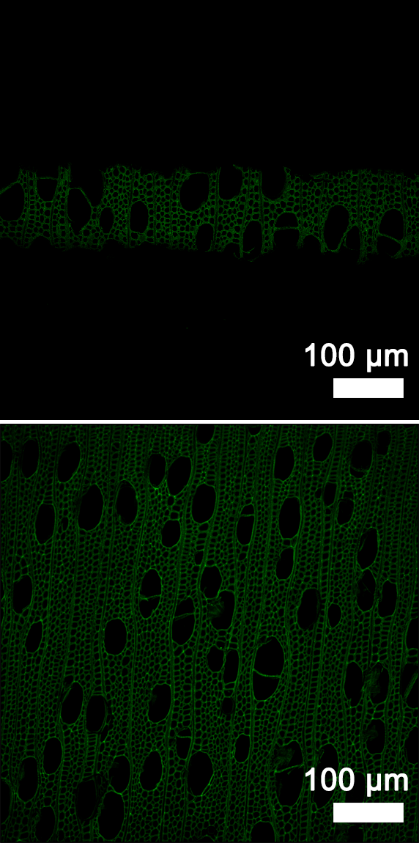


**Figure S5.** The LSCM images of the 6 mm thick poplar wood veneer.


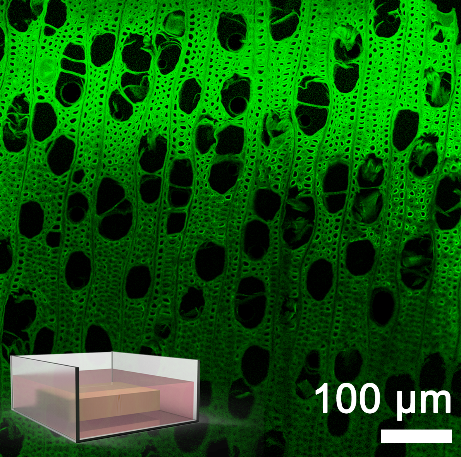


**Figure S6.** LSCM images of poplar veneer with thickness of 6 mm following impregnation with low-molecular-weight phenolic resin.


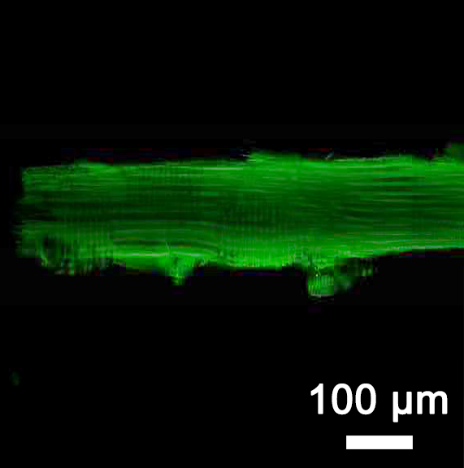


**Figure S7.** LSCM images of poplar veneer with thickness of 0.2 mm after impregnation in MUF resin with added fluorescent agent.


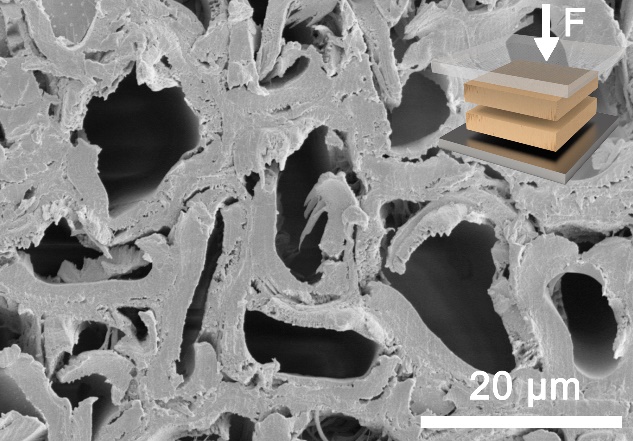


**Figure S8.** SEM images of stacked poplar veneers with thickness of 6 mm and hot pressed.


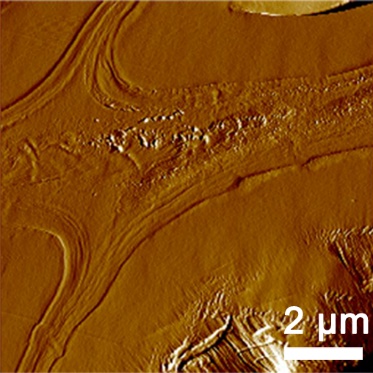


**Figure S9.** The morphologies images of untreated poplar veneer measured by AFM (PF-QNM).


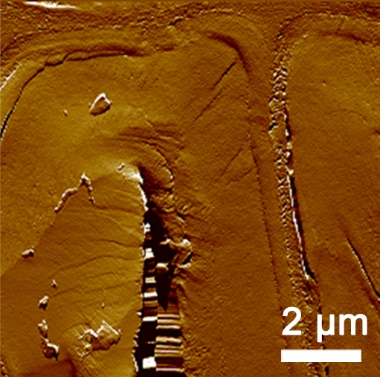


**Figure S10.** The morphologies images of poplar veneer after impregnation and curing with phenolic resin measured by AFM (PF-QNM).


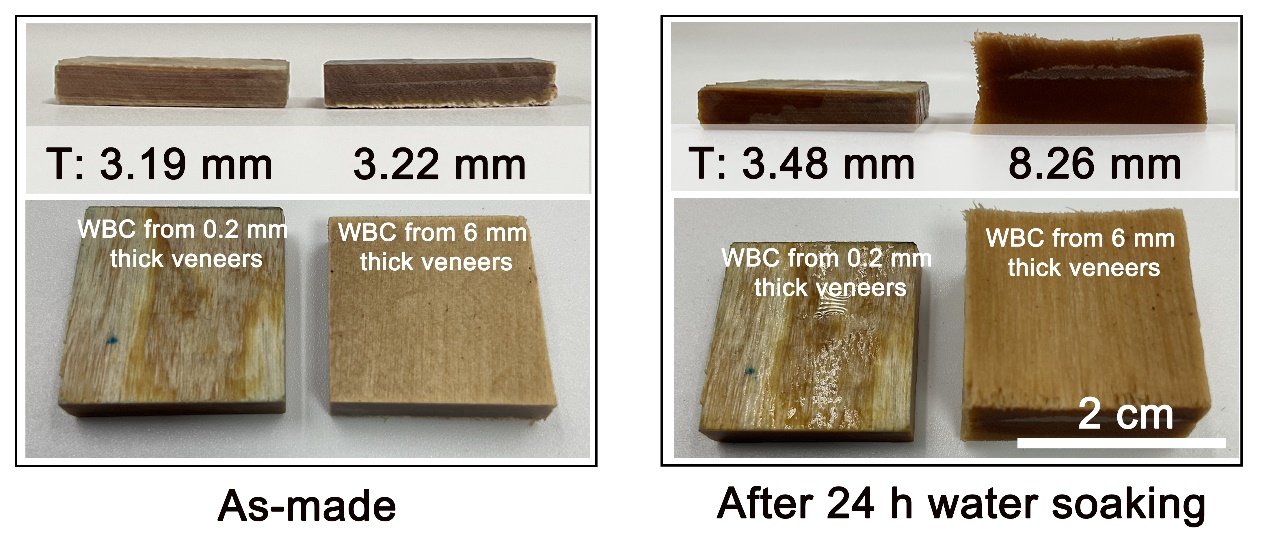


**Figure S11.** A comparison of WBC from 0.2 mm and 6 mm thick veneers before and after 24 h water soaking.


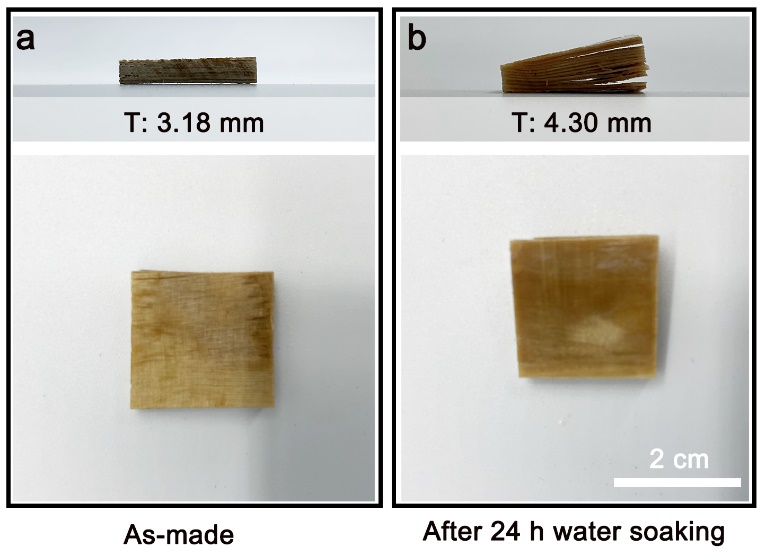


**Figure S12.** A comparison of WBC with thickness of 0.2 mm following impregnation with MUF resin before and after 24 h water soaking.


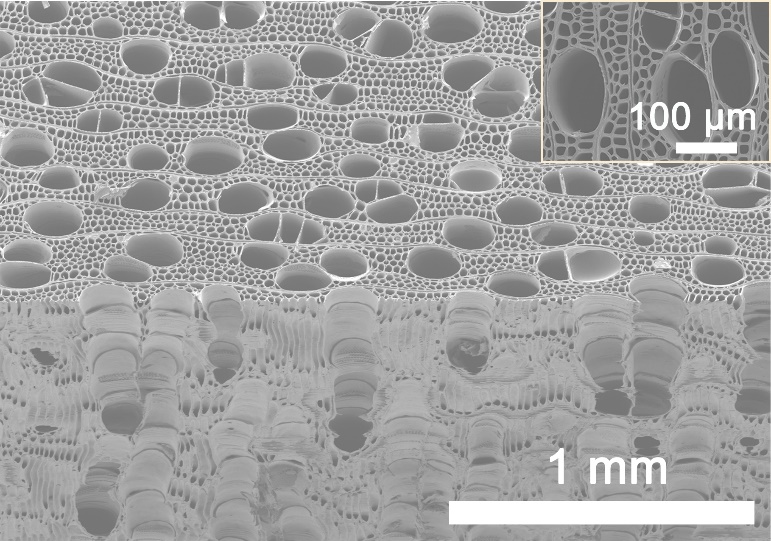


**Figure S13.** Two-section SEM images of the poplar wood.


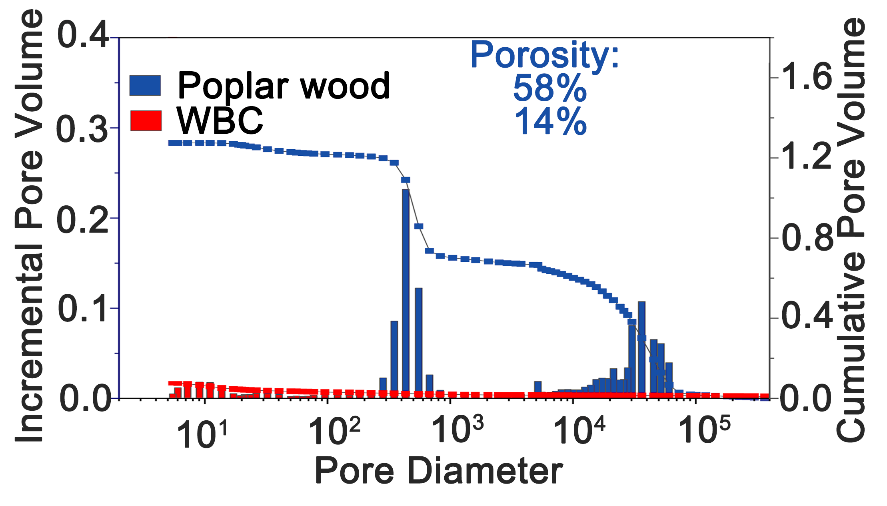


**Figure S14.** The Incremental Pore Volume, Cumulative Pore Volume and porosity of the poplar wood and the super-hard wood composites.


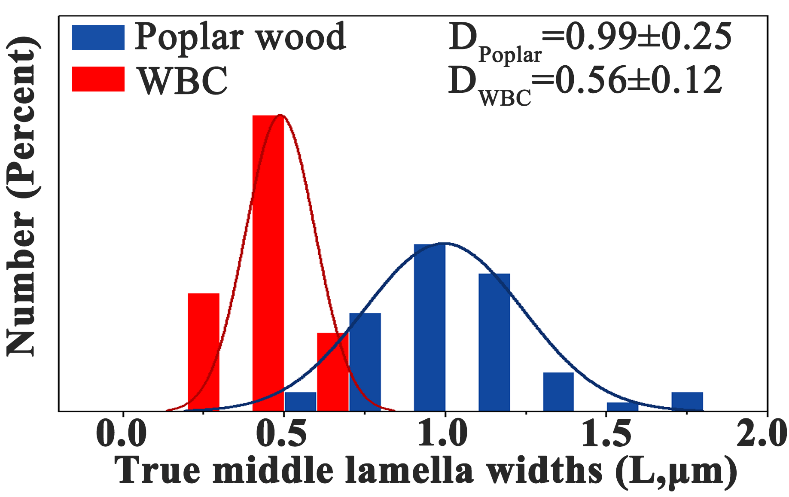


**Figure S15.** Middle lamella widths size distributions of the poplar wood and WBC.

**Figure S16.** FTIR spectra of poplar wood, PF resin and WBC. It reveals structural compatibility between lignin and PF resin. The preservation of lignin’s aromatic vibrations (1596, 1459 cm⁻¹) and cellulose’s hydroxyl groups (3325 cm⁻¹) confirms non-destructive integration, while methylene bridge formation (880 cm⁻¹) validates PF self-curing.


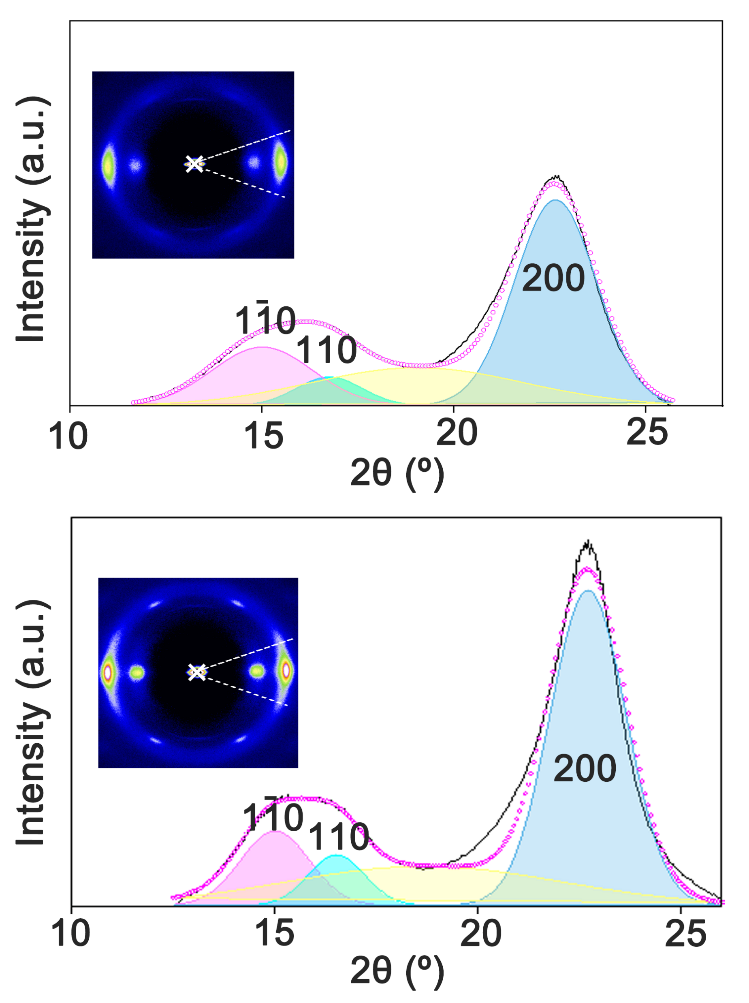


**Figure S17.** The 2D-WAXD patterns of the poplar wood. The crystallinity of the samples was calculated by the ratio of peak intensity in the crystalline area to the total integrated intensity of each diffraction peak.


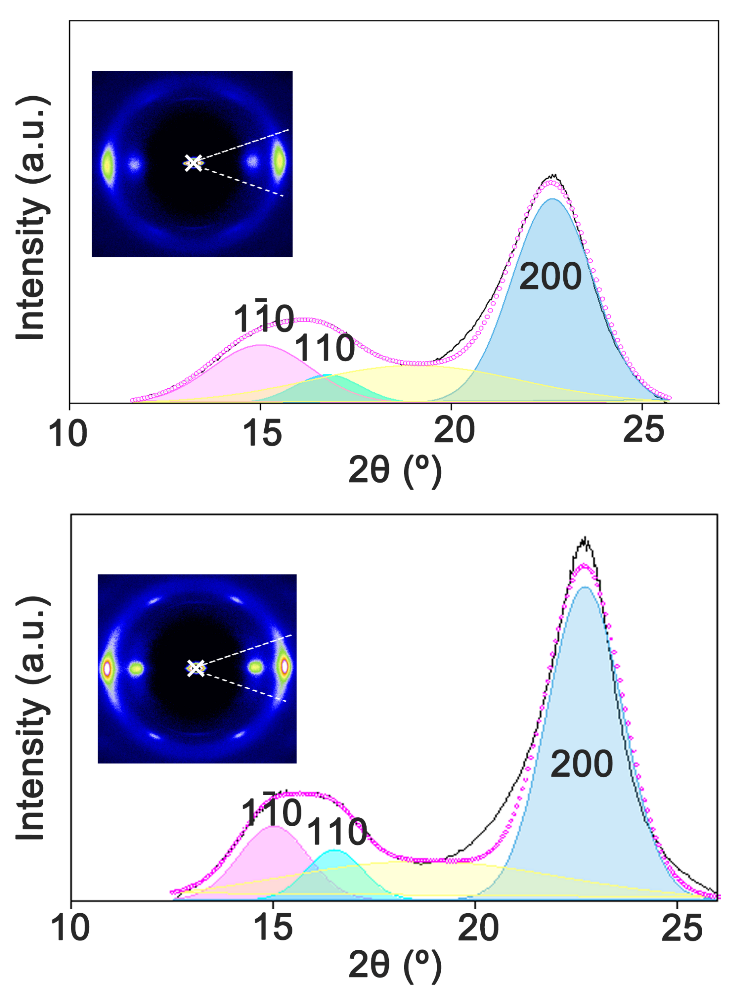


**Figure S18.** The 2D-WAXD patterns of WBC.


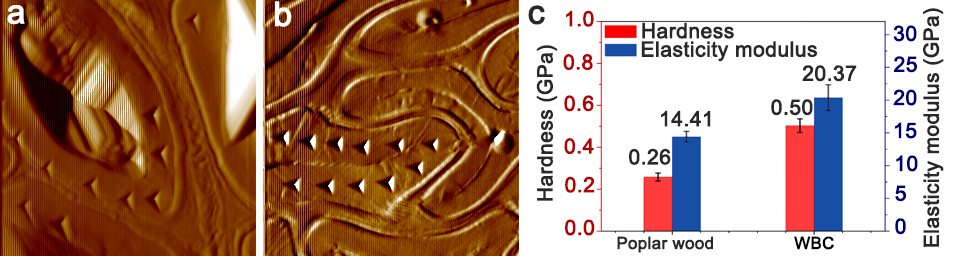


**Figure S19.** NI images of poplar wood (a) and WBC (b), (c) The cell wall hardness and elastic modulus of the poplar wood and super-hard wood composites.


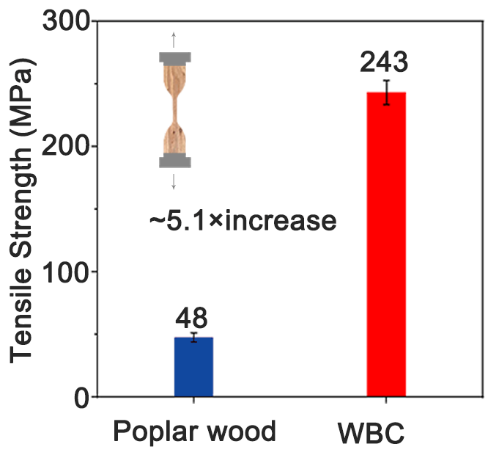


**Figure S20** Tensile strength of the poplar wood and WBC.


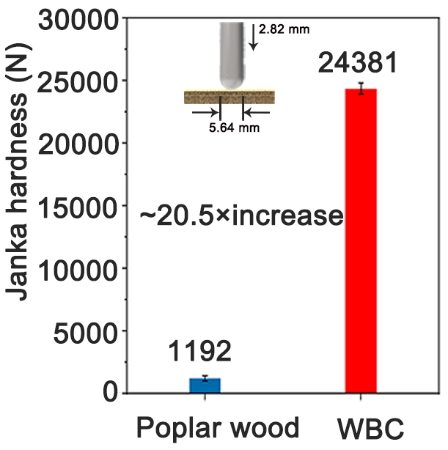


**Figure S21** Janka hardness of the poplar wood and WBC.


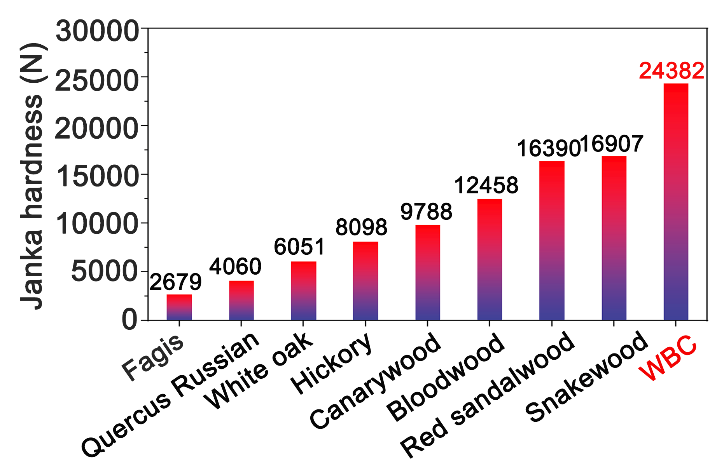


**Figure S22** Comparison of the Janka hardness of WBC with most high-quality hardwood.


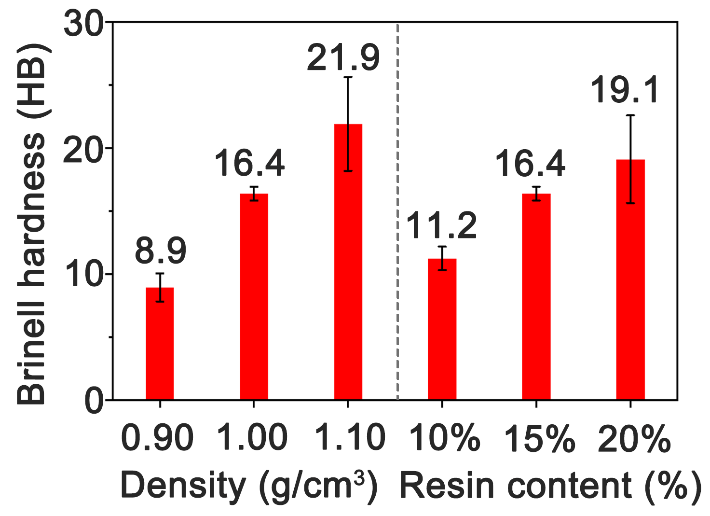


**Figure S23** Brinell hardness of WBC with different densities and resin contents**.**


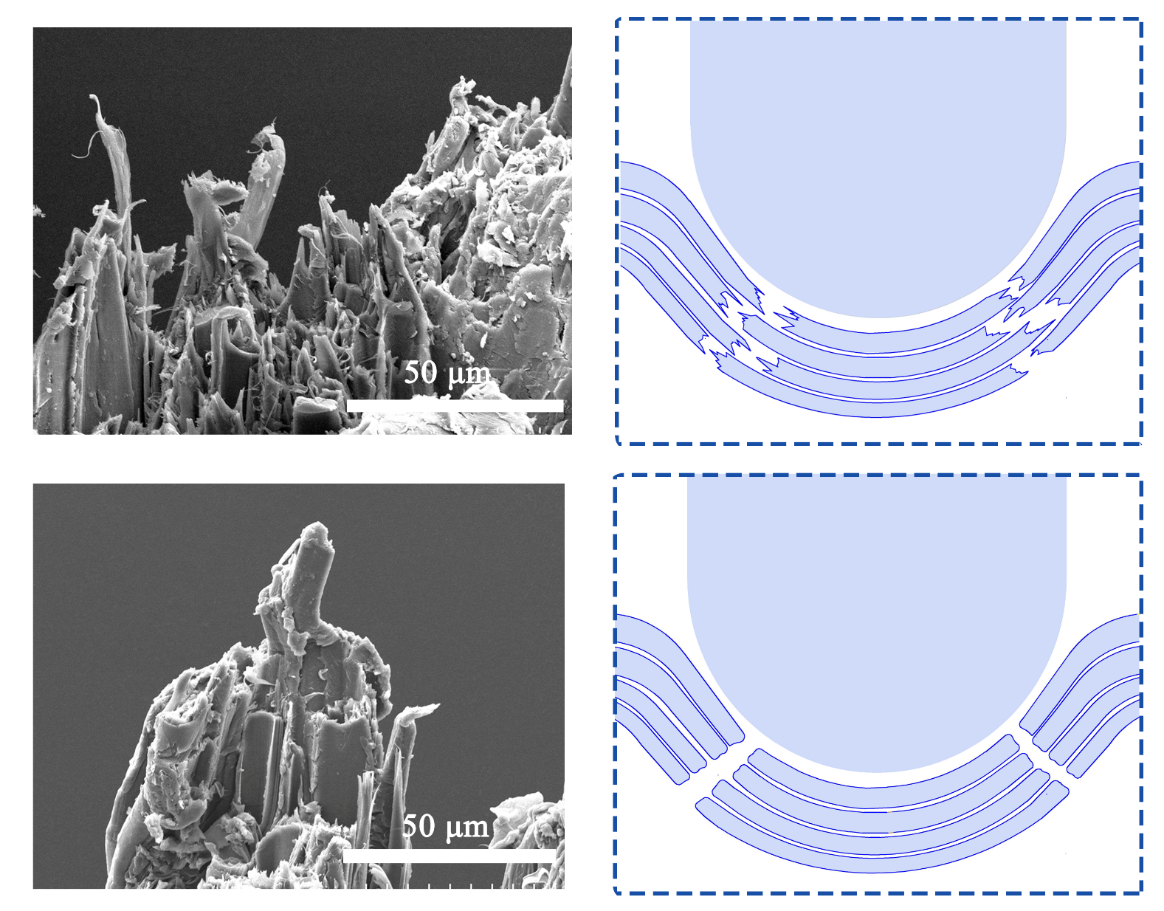


**Figure S24** Schematic diagram of poplar wood after hardness damage.


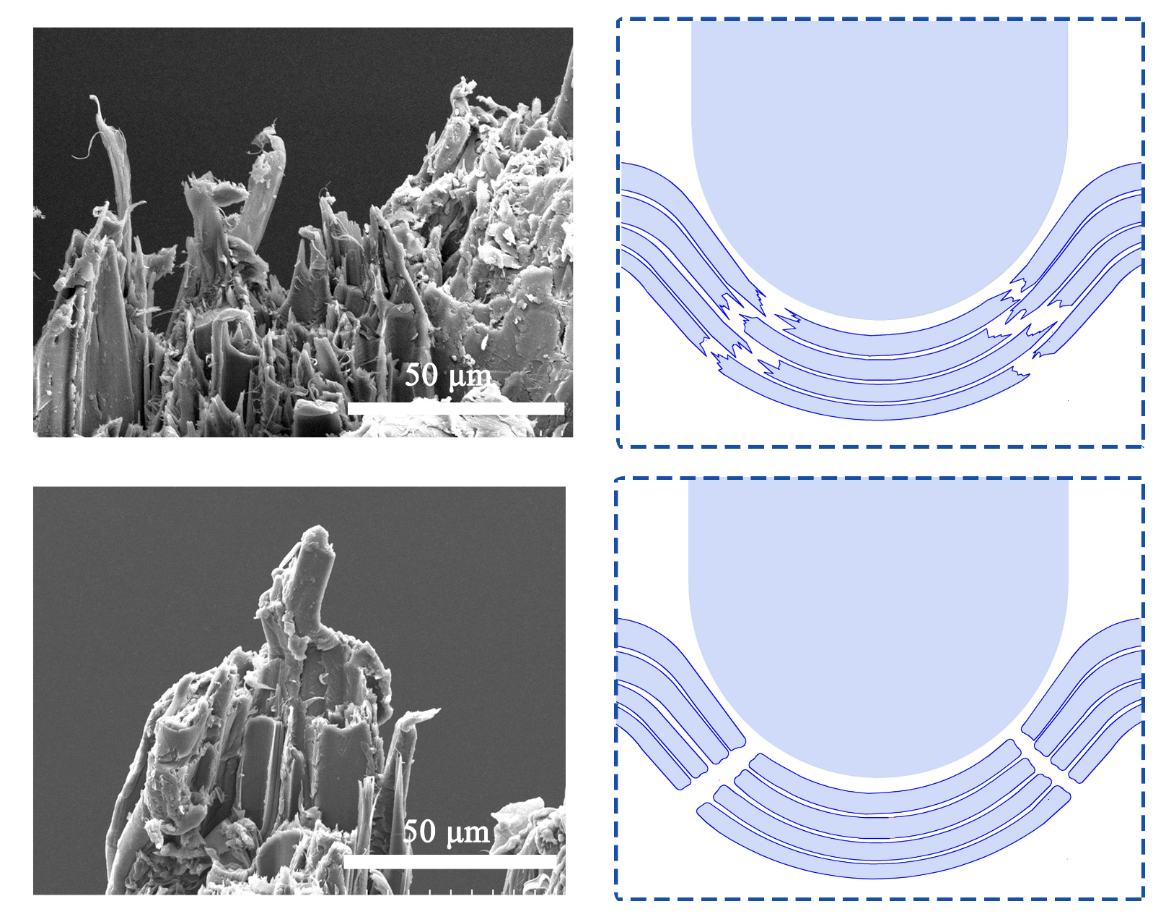


**Figure S25** Schematic diagram of WBC after hardness damage.


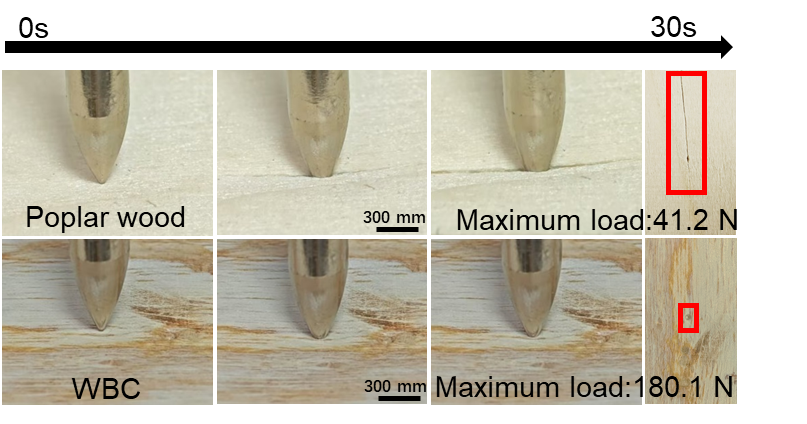


**Figure S26** Schematic diagram of static puncture test for poplar and WBC.


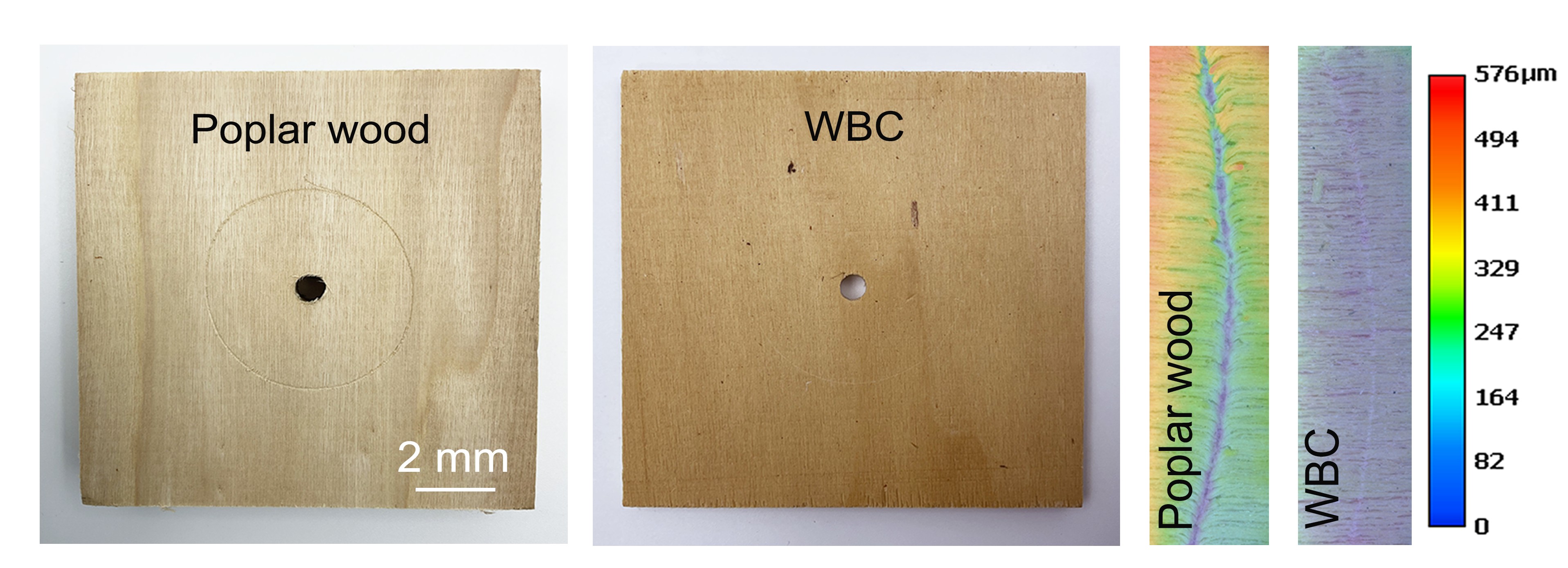


**Figure S27** Schematic diagram of scratch resistance test on poplar and WBC surfaces.

**Figure S28** TSR during combustion of the poplar wood and WBC.

**Figure S29** THR during combustion of the poplar wood and WBC.

**Figure S30** Intact grade of poplar wood and WBC.


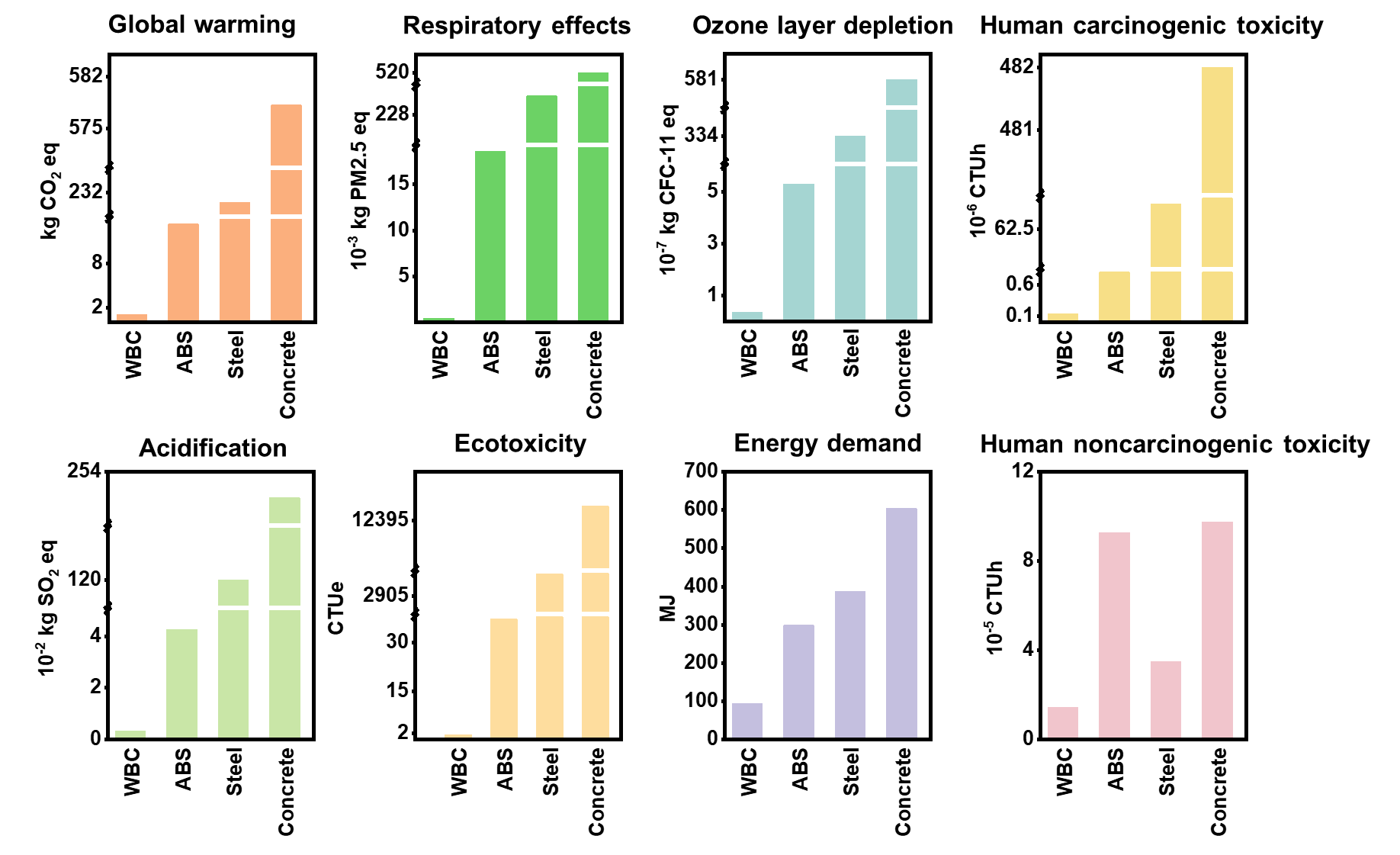


**Figure S31** Comparative assessment of the environmental impacts of WBC, ABS, steel and concrete.

**Table S1**. Comparison of properties of WBC, engineering plastics and steel.

|  | WBC | Engineering plastics (polyoxymethylene) | Steel (Stainless steel SUS316L) |
| --- | --- | --- | --- |
| Strength (MPa) | 305 | 107 | 205 |
| Hardness (HB) | 40.7 | 11.2 | 161 |
| Modulus (GPa) | 32 | 2.75 | 193 |
| Density (g·cm^-3^) | 1.35 | 1.40 | 7.98 |
| Specific strength (MPa/(g·cm^-3^)) | 226 | 76.4 | 26 |
| Specific hardness (HB/(g·cm^-3^)) | 30.15 | 8.0 | 20.14 |
| Specific modulus  (GPa/(g·cm^-3^)) | 24 | 1.96 | 24 |
| 1/Density (cm^3^ ·g^-1^) | 0.74 | 0.71 | 0.13 |
| Price (yuan/kg) | 2.9 | 30.0 | 5.0 |
| Cost-effectiveness (kg yuan^-1^) | 0.34 | 0.03 | 0.2 |

**Table S2**. The multiple of WBC, ABS, steel, and concrete in terms of environmental impact (normalized to the most impact substances in the environmental impact category).

| Impact category | WBC | ABS | Steel | Concrete |
| --- | --- | --- | --- | --- |
| GWP (kg CO_2_ eq) | 0.0019 | 0.0230 | 0.3988 | 1 |
| ET (CTUe) | 0.0001 | 0.0030 | 0.2348 | 1 |
| ODP (kg CFC-11 eq) | 0.0006 | 0.0092 | 0.5749 | 1 |
| AP (kg SO_2_ eq) | 0.0013 | 0.0168 | 0.4743 | 1 |
| PED (MJ) | 0.1554 | 0.4945 | 0.6404 | 1 |
| RI (kg PM2.5 eq) | 0.0009 | 0.0358 | 0.4423 | 1 |
| HT-cancer (CTUh) | 0.0003 | 0.0017 | 0.1305 | 1 |
| HT-non cancer (CTUh) | 0.1476 | 0.9514 | 0.3566 | 1 |

**Table S3.** The formaldehyde emission of poplar wood and super-hard wood composites.

|  | Formaldehyde emissions (mg/m^3^, 1 m^3^ climate box method) | |
| --- | --- | --- |
|  | Poplar veneer | super-hard wood composites |
| Detection value | 0.014 | 0.007 |

**Table S4.** The TVOC release rate (72 h, mg/ (m^2^·h)) of poplar wood and super-hard wood composites.

| Materials | The TVOC release rate (72 h, mg/ (m^2^·h)) | | |
| --- | --- | --- | --- |
|  | Standard specified value | Detection result | Decision result |
| super-hard wood composites | ≤0.05 | 0.01 | Eligible |
